# Säntis lightning research facility: a summary of the first ten years and future outlook

**DOI:** 10.1007/s00502-022-01031-2

**Published:** 2022-04-22

**Authors:** Farhad Rachidi, Marcos Rubinstein

**Affiliations:** 1 EMC Laboratory, EPFL-SCI-STI-FR, Station 11, Swiss Federal Institute of Technology (EPFL), 1015 Lausanne, Switzerland; 2 Institute for Information and Communication Technologies, University of Applied Science and Arts Western Switzerland, Yverdon-les-Bains, Switzerland

**Keywords:** Lightning, Lightning measurements, Lightning protection, Lightning currents, Electromagnetic fields, Blitz, Blitzmessungen, Blitzschutz, Blitzströme, Elektromagnetische Felder

## Abstract

The Säntis Tower was instrumented in May 2010 to measure currents of lightning discharges striking the structure. Since then, the system has been recurrently updated and expanded. Presently, data associated with lightning striking the tower are collected at six different sites. The facility is equipped with a current measurement system, three electric field stations, an electrostatic field mill, two x‑ray sensors, a high-speed camera, and four slow cameras. This paper presents the latest measurement configuration at the facility. Other temporarily loaned instruments are also briefly described. Furthermore, examples of some of the data that have been gathered and analyzed are given, and an outlook as well as future plans for the facility are presented.

## Abstract (de)

Der Säntisturm wurde im Mai 2010 mit Messinstrumenten ausgerüstet, um Ströme von direkten Blitzentladungen aufzunehmen. Seitdem wurde das System immer wieder aktualisiert und erweitert. Derzeit werden Daten im Zusammenhang mit Blitzeinschlägen in den Turm an sechs verschiedenen Standorten aufgezeichnet. Die Anlage ist mit einem Strommesssystem, drei elektrischen Feldstationen, einer elektrostatischen Feldmühle, zwei Röntgensensoren, einer Hochgeschwindigkeitskamera und vier Kameras mit einer Standard-Bildrate ausgestattet. In diesem Beitrag wird die neueste Messkonfiguration der Anlage vorgestellt. Auch andere, vorübergehend ausgeliehene Instrumente werden kurz beschrieben. Weiters werden Beispiele der gesammelten und analysierten Daten sowie ein Ausblick und Zukunftspläne für die Anlage präsentiert.

## Introduction

Lightning is a cause of deleterious effects on electronic equipment, infrastructure, forests and it is also responsible for loss of human life and livestock [1]. The risk of death to humans has decreased, mostly in developed countries, due to protective measures and to risk awareness and knowledge of the appropriate course of action when thunderstorms approach. However, other risks, such as, for instance, the risk of disturbance and damage of power generation, transmission and distribution systems, are not only still present, but they are increasing as renewable energy sources and smart-grid control electronics continue to be integrated into power systems (e.g., [2]). In addition to the increase in the number of potential victims of disturbance, lightning itself may become more energetic and frequent due to the influence of climate change on weather phenomena [3, 4].

It is therefore important, given the issues that were just outlined, to develop optimize and appropriate lightning protection strategies against the responsible lightning processes using quality lightning data. The knowledge of the lightning channel-base current is of great importance because it can be used as a basis to study the effects of and the protection against direct strikes and indirect electromagnetic effects.

**Fig. 1:**
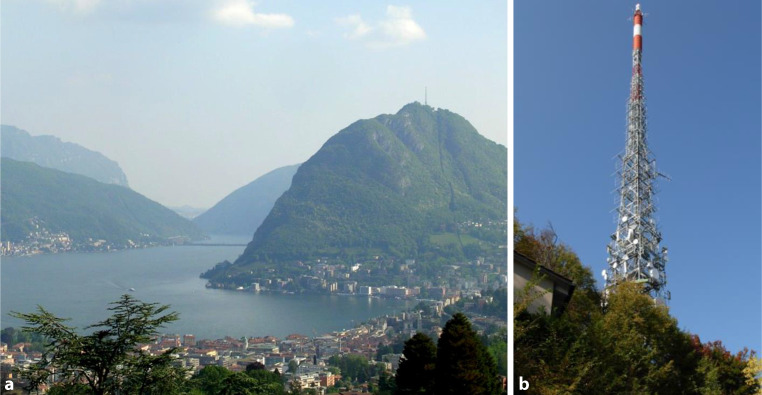
View of San Salvatore Mountain (**a**) and the tower (**b**) on its summit

Our current knowledge of lightning current parameters comes essentially from direct measurements obtained using instrumented towers and from artificially initiated lightning (e.g., [5]). Extensive experimental data recorded by Prof. Karl Berger and his team on the top of two instrumented towers in Monte San Salvatore in Southern Switzerland from the 1950s through the 1970s [6] resulted in a comprehensive statistical characterization of lightning current parameters. Mount San Salvatore has a height of 640 m above the level of the adjacent Lake Lugano and it is 914 m above sea level. The first tower for lightning measurements was constructed on the summit of San Salvatore Mountain in 1943. It was replaced by a radio and television tower in 1958, on which the measurement of lightning discharges continued. In 1950, a second lightning research tower was constructed 400 m to the North from the first one. Both towers were 70 m tall. Later, the second tower was demolished and nowadays only the radio and television tower is present on the summit of the mountain as shown in Fig. 1. Lightning currents were measured by means of a two-stage shunt just below the needle of each tower and recorded by electromagnetic and cathode ray oscillographs. On each tower, two different shunts were used in series, one with a resistance of 0.05 Ω for currents in the 1 kA to 200 kA range and a the other with a 0.8 Ω resistance used for currents from 50 A to 24 kA [6].

The work of Prof. Berger and the data obtained have been instrumental in our understanding of many of the processes in the lightning discharge and in the classification of lightning into its various types. The results of Prof. Berger and his team were obtained using instrumentation that, although modern at the time, had a frequency bandwidth limited to some hundreds of kHz and, due to the limited time of each recording, did not include the full lightning time span.

**Fig. 2:**
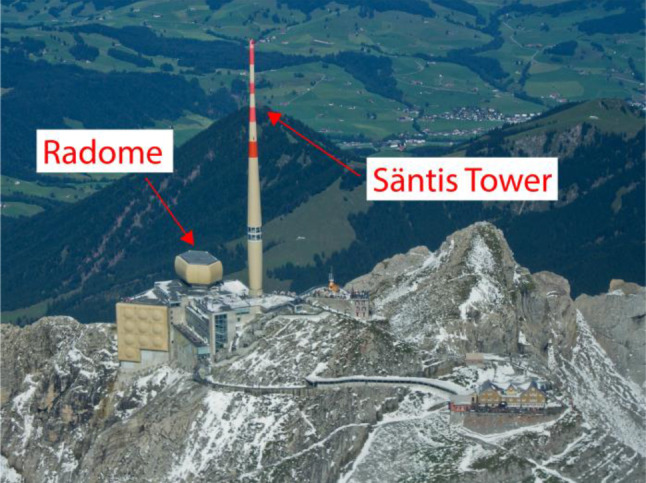
The Säntis Tower and the radome that houses electromagnetic field, x‑ray, and sound sensors. Rogowski coils and B‑dot sensors are installed inside the tower hull, at 24 m and 82 m

The Säntis Tower in Northeastern Switzerland, which is the subject of this paper, was identified as an ideal candidate for lightning measurements and it was instrumented for that purpose thanks to several research projects funded since 2009 by the Swiss National Science Foundation, the State Secretariat for Education and Research, and the European COST Action P18. A picture of the Säntis Tower is shown in Fig. 2.

The Säntis Tower is 123.5 m tall. It sits on top of the 2502 m tall Mount Säntis, which is the tallest mountain of the siliceous limestone sedimentary rock Alpstein mountain complex [7]. The tower has an inner metal structure of 2.5 m mean radius with an outer Plexiglas structure of 6 m mean diameter. The base of the tower has a diameter of 8 m and it rests on a set of metal supports that allows the structure to sway slightly under heavy winds.

Several towers of increasing length, each replacing the previous one, were erected throughout the years at the same site, starting with a weather station in 1880, followed by an 18‑m tall TV antenna installed in 1958, which was itself replaced in 1976 by an 84 m tall structure that served as a telecommunications tower, and which was superseded in 1997 by the current tower. Interestingly, the thunder day count at the site exhibited step-like increases every time a taller tower replaced the previous one. The opposite was also observed during the periods when a tower had been dismantled to construct the new one. The current structure is a telecommunications tower operated by Swisscom Broadcast and the site is also used as a weather station.

**Fig. 3:**
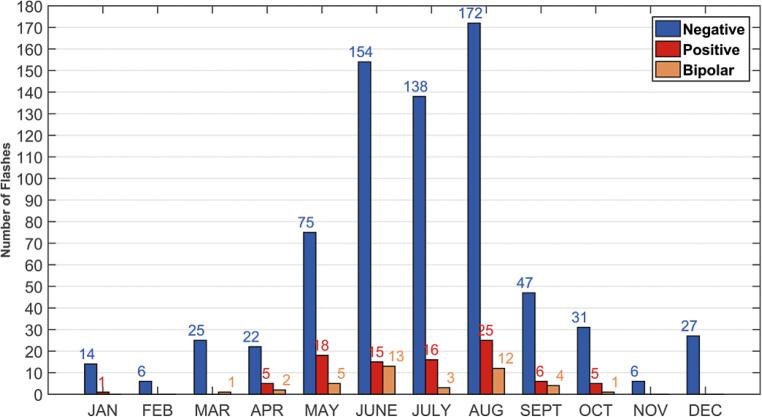
Monthly distribution of recorded flashes at the Säntis Tower

The Säntis tower is consistently struck by lightning some 100 times a year. This makes the tower a steady source of direct experimental lightning data. In the first ten years of operation of the station, nearly 1000 flashes were recorded and analyzed. The majority (more than 95%) of the flashes were of upward type. The analysis of part of the data can be found in [8]. Over the period from May 2010 to mid-August 2019, a total of 849 flashes were recorded. About 11% of the recorded flashes (91) were positive and about 5% (42) were classified as bipolar. The remaining 716 flashes were all negative. A histogram of the monthly distribution of the number of flashes to the Säntis Tower until August 2019 is shown in Fig. 3. As can be seen, the lightning activity spreads over the whole year with a clear concentration for both negative and positive flashes during the summer months, August being the month during which most of the lightning occurred (172 negative and 25 positive). Except for the months of February and November, the average number per month is always higher than 1, this average being above 10 in May, June, July and August.

## Säntis Tower instrumentation

The Säntis Lightning Research Facility has been operational since 2010 and it currently includes an expanding array of sensors as well as remote monitoring and control capabilities. The permanent as well as temporary instruments used at the facility are presented in the next subsections.

### Overall facility and measurement setup

**Fig. 4:**
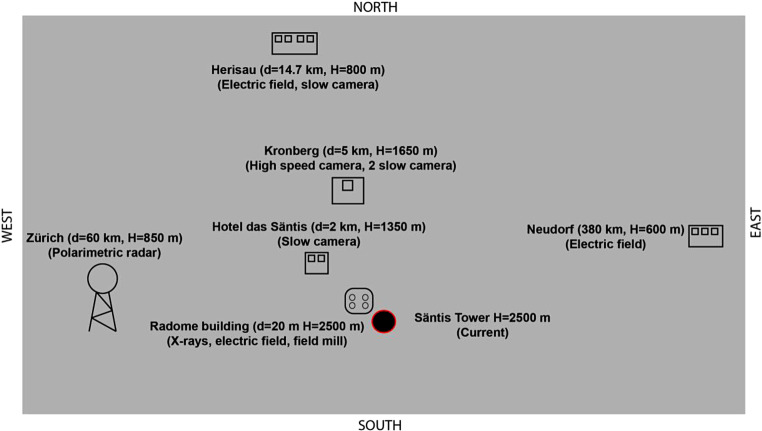
Simplified sketch of six different observational sites and measuring sensors including their distance d to the tower and their geographical altitude H. Not to scale

Fig. 4 presents a simplified sketch of the measurement sites belonging to the Säntis research facility. The measurement systems deployed in each site are briefly described in what follows.

### The Säntis Tower (2502 m ASL): lightning current measurements

**Fig. 5:**
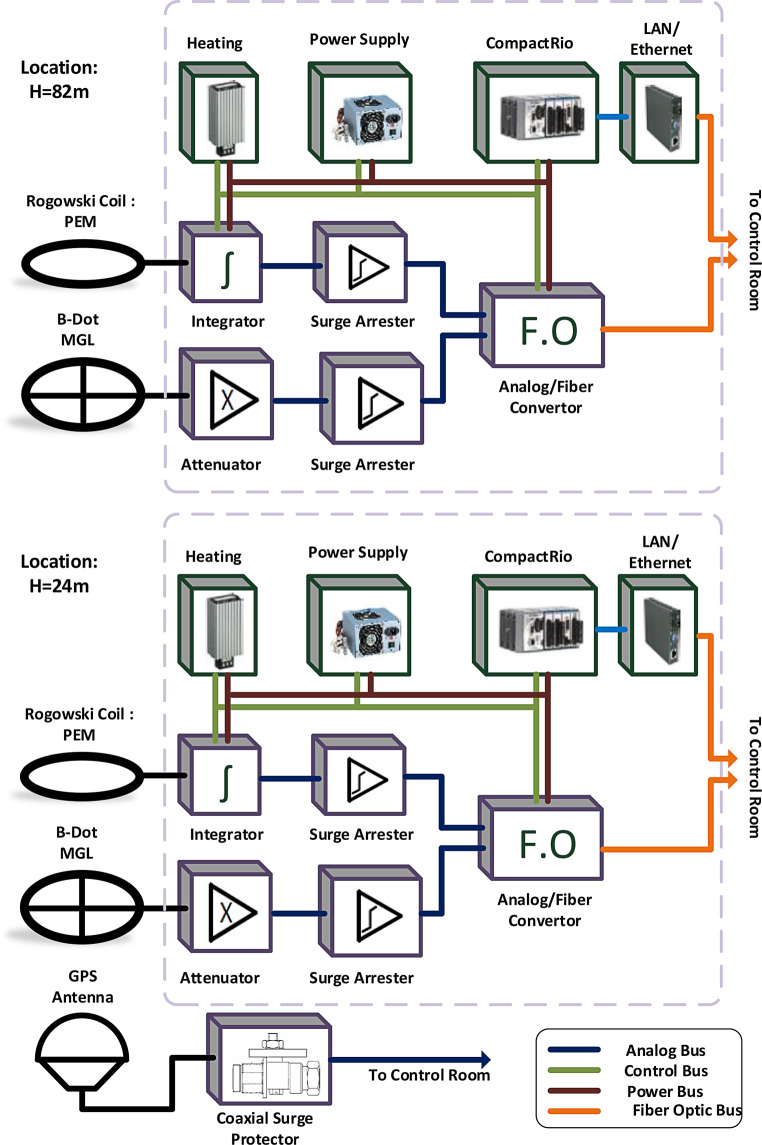
Schematic diagram of the current measurement system at Säntis. (Adapted from [13])

When the Säntis Tower was first instrumented in 2010, sensors to record the lightning current waveforms and their time-derivatives were installed. The currents and current derivatives are presently measured at two different heights, 24 m and 82 m AGL, using, at each height, a Rogowski coil and a multigap B‑dot sensor. The specially built multigap B‑dot sensors, which are placed against the outside of the core mast of the tower to measure signals proportional to the derivative of the lightning current, have a 20-MHz bandwidth. They are used to acquire the faster parts of the lightning current since the Rogowski coils have a limited high frequency response. The B‑dot sensors are based on the proposed design published in the 1960s by Baum, Breen, Giles, O’Neill, and Sower [9, 10]. Fig. 5 presents the schematic diagram of the lightning current measurement system. The measurement systems on the tower are thoroughly described in [11–13]. The measured signals are transmitted over a fiberoptic link to a National Instruments PXI-5122 high-speed digitizer set to record each detected lightning flash with a measurement window of 2.4 s at a sampling rate of 50 MS/s [8, 14].

**Fig. 6:**
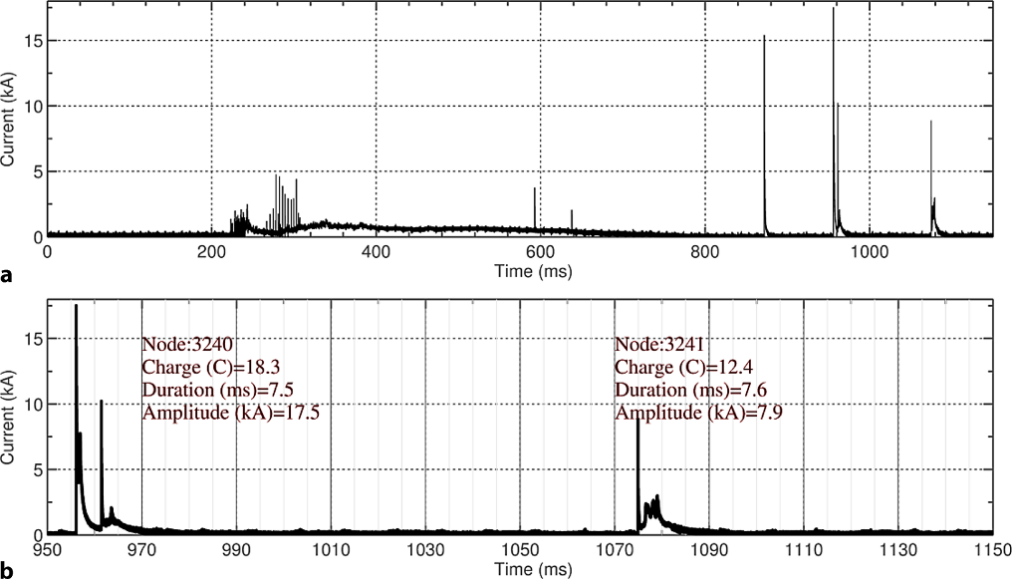
Example of a current waveform associated with an upward negative flash occurred on 2011-07-13 at 17:36.26. **a** Overall flash current. **b** Details of the last two return strokes

Fig. 6 presents an example of a current waveform associated with an upward negative flash. The presented waveform was measured by the Rogowski coil located at 82 m. The current waveform is typical of upward negative flashes with an initial continuous current (ICC) of about 400 ms duration and superimposed ICC pulses. After the extinction of the ICC, four return strokes occurred, the second being characterized by the highest peak, with an amplitude of about 20 kA. The overall charge transferred to ground by this flash was close to 200 C.

**Fig. 7:**
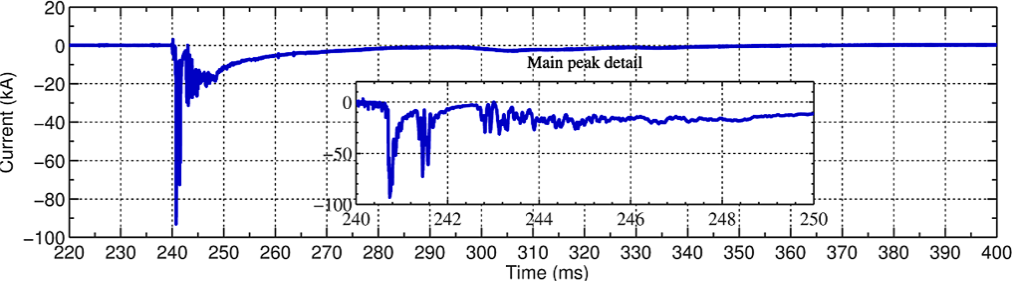
Current waveform associated with a positive flash that occurred on August 3, 2011 at 11:51. An expanded view of the first 10 ms of the waveform is presented in the inset of the figure

Fig. 7 shows an example of a current waveform associated with a positive flash. Note that the waveform is characterized by a complex waveshape including two pulses (of peak amplitudes 93 kA and 72 kA, respectively) separated by 1 ms, and followed by a series of fast pulses superimposed on a long continuing-current-like waveform. The total charge transferred to ground for this particular flash was in excess of 400 C.

### Radome (2502 m ASL): E-field mill, fast E-field, X-ray, microphone

The radome is located 20 m away from the tower (see Fig. 2). An industrial PC with a two-channel PCI digitizer is set up inside the dome. One channel of the digitizer is connected to a fast E‑field antenna and the second is connected to one of two X‑ray sensors. The sampling rate is set to 50 MS/s with a pre-trigger delay of 1.2 s. Each record is 2.4 s long. A field mill is connected to the same PC. A Garmin GPS 18x LVC is connected to the serial port of the industrial computer and it provides a time accuracy of several microseconds. A commercial microphone is also installed and connected to the PC with a USB cable. It is operated via LabView and it provides sound recordings for a duration of 100 s. The data are saved only when a trigger is received. Information about the sensors is given in what follows.

#### Field mill

**Fig. 8:**
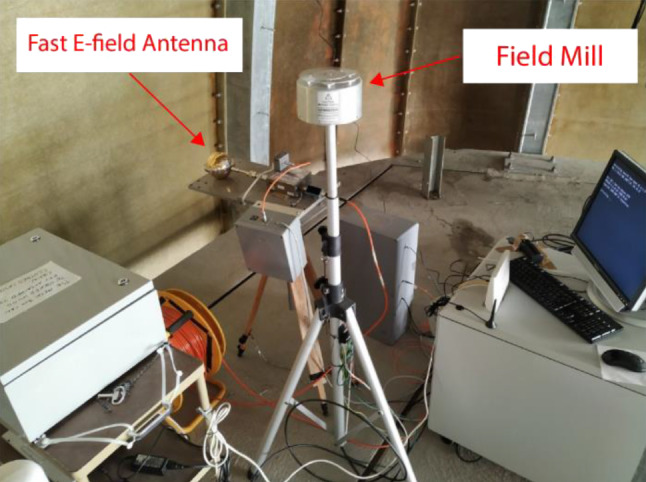
Fast E‑field antenna and field mill

This electrostatic field sensor is an EFM-100 field mill installed since July 15, 2016 as shown in Fig. 8. The field mill is connected using a USB cable to the industrial PC and the data are recorded in continuous mode.

#### Fast antenna

A commercial Mélopée electric field sensor with a frequency range of 1 kHz to 150 MHz was installed during the Summer of 2018 (see Fig. 8). The signal from this antenna is relayed by an optical link to a receiver, the output of which is connected to the first channel of the digitizer.

#### X-ray sensors

**Fig. 9:**
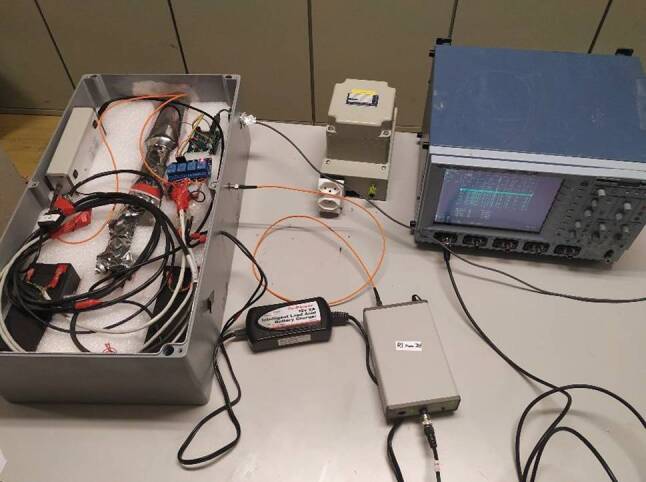
X‑ray sensor from Uppsala University

In July 2019, an X‑ray sensor (see Fig. 9) from Uppsala University [11] was installed in the radome. In order to mitigate the coupling and interference of strong lightning electromagnetic fields to the measuring system, a battery power supply was installed in the metallic box containing the X‑ray measuring device. This system consists of two batteries and a microcontroller that manages the charging of the batteries in such a way that the charging alternates between the two. While one battery is being charged, the other is used as the power supply, so that the system is never connected galvanically to the 230 V grid, preventing any conducted interference from reaching the equipment. Furthermore, the 230 V power supply is provided from an insulation transformer to further reduce noise in cables and possible field coupling. To further reduce the noise, the analog output of the X‑ray sensor is relayed to the second channel of the digitizer via a fiberoptic link.

**Fig. 10:**
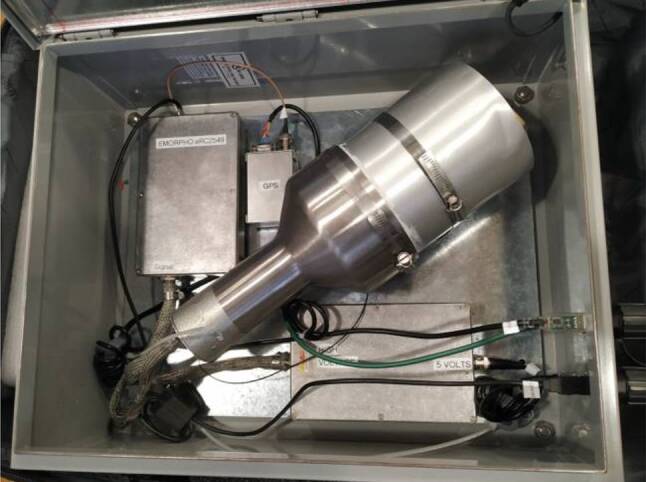
X‑ray sensor from the University of California, Santa Cruz working in the continuous mode

Another X‑ray sensor belonging to the University of California, Santa Cruz (see Fig. 10) was also installed in July 2019. The detector is a 5-inch (diameter) × 5-inch (length) cylinder of BC-408 plastic scintillator mounted to a 5-inch PMT (photomultiplier tube). The PMT is negatively biased by ~ 850 Volts. The detector is connected to a Bridgeport Instrument eMorpho MCA (Multi-Channel Analyzer) that uses a time-tagged event mode to record the integrated pulse area (with 16-bit resolution) and the arrival time (with 32-bit/12.5-ns resolution) of the detector output. This amounts to a 40 MHz sampling speed. The combination of the nanosecond decay time of the BC-408 scintillator and the sampling speed of the MCA is needed to record the high flux and sub-millisecond arrival times of terrestrial gamma-ray flash (TGF) photons. In addition to the detector chain, there is also a GPS unit where a pulse-per-second signal is fed into the MCA’s FPGA and incorporated into the data stream as a flagged event. This allows for a precise relative timing and low data usage since the periods without events are not saved, so the device can operate in continuous mode. This device is connected to a second computer on which the data are saved.

#### Microphone

A commercial microphone was installed in August of 2019. The frequency response of the microphone is relatively flat in 20 Hz to 20 kHz range. The microphone has a supercardioid radiation pattern. It is connected via USB to the industrial PC.

### Das Säntis Hotel: slow camera

Das Säntis Hotel (1400 m ASL) is located about 2 km away from the tower and 1150 m below the top of the mountain, on the slope of Mount Säntis. In Spring 2020, a slow camera, consisting of a Raspberry PI 4 with a Camera Module v2 was installed in the hotel. The camera has an optical size of 1/4″ and a focal ratio equal to 2.0. It is currently set to an ISO equal to 100 and is operating in Full HD resolution with 30 FPS.

The Raspberry PI is equipped with a 32 GB SD card and it is connected to the Internet. The main code is written in Python and it consists of two threads. The first waits for the trigger over TCP/IP on the chosen port. The second records video continuously on a circular memory for a duration of 60 s. If a trigger is received, the video is saved. Otherwise, the video is deleted. Remote access of the raspberry PI is possible over its Internet connection.

### Kronberg, Mount Kronberg (1663 m ASL): high-speed video

**Fig. 11:**
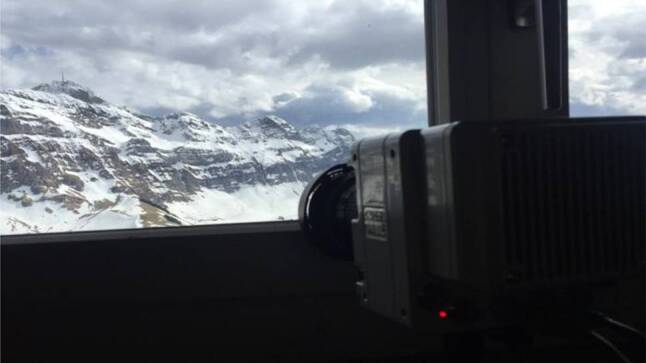
High speed Phantom VEO 710L camera installed at the top of the Kronberg mountain

Mount Kronberg is about 4 km away from the tower, direction North. A Phantom VEO 710L high-speed video camera is installed at this location. A view of the Säntis Tower from the camera is shown in Fig. 11. The camera can record up to 1 MFPS at its lowest resolution of 8 × 8 pixels. To have a wider view of 512 × 512 pixels, the number of frames per second was reduced to 10,000. These pixels are distributed over a view of about 2 km by 2 km. The camera records during a 3-second time window with a pre-trigger delay of 1.5 s. A GPS time stamp is provided with an Acutime 360 Multi-GNSS Smart Antenna and the synchronization error is within 15 nanoseconds. Additionally, two slow cameras identical to the one described in the previous subsection are installed at this location. The first camera works in the visible spectrum and the second one operates in infra-red.

An example of high-speed video for an upward negative lightning flash recorded on June 18, 2019 can be seen in this link: https://www.youtube.com/watch?v=lakaE-fKQf4&feature=emb_title.

### Herisau (800 m ASL): fast E-field measurements

**Fig. 12:**
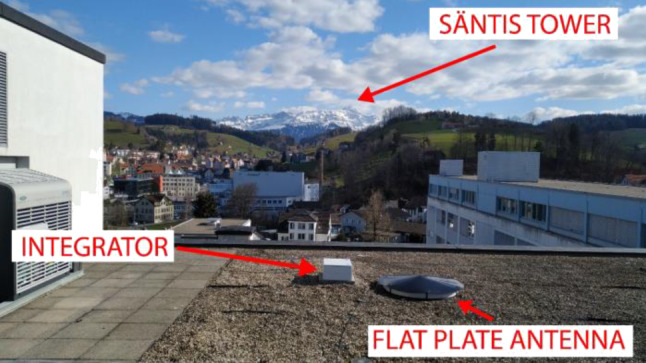
Flat plate antenna installed at the Herisau site. The antenna is covered with a dielectric for precipitation protection

This site is located at a distance of 14.7 km from the tower. A flat plate antenna was installed in this location in 2017 and it has been updated several times since then (see Fig. 12). The fast E‑field sensor uses a two-battery system that switches to one of the batteries for regular operation while the other battery is charging off the mains, thus avoiding having a galvanic connection to the mains during the normal sensor operation as in the case of the X‑ray sensor (Subsection 2.3). An industrial computer with a two-channel digitizer is installed at the station. The antenna is connected to the first channel that can measure voltages from −5 V to 5 V with a 14-bit resolution. The time constant of the antenna is 8 ms and it can measure electric fields in the range from −200 V/m to 200 V/m. The digitizer is set to a sampling rate of 10 MS/s with a time window of 6 s and a pre-trigger delay of 3 s. The second channel of the digitizer is connected to the pulse per second output of a Garmin GPS 18x LVC unit. This provides a time accuracy of about a microsecond. This site is also equipped with one slow camera with the same specifications described in Subsection 2.4.

### Neudorf, Austria: fast E-field measurements

In addition to the close (Radome) and intermediate (Herisau) field sensors, the wideband vertical electric field is also measured in Northwestern Austria, at a distance of 380 km, by way of a wideband electric field sensor belonging to ALDIS (Austrian Lightning Detection and Information System) in Vienna. The electric field sensor consists of a flat plate antenna and an integrator with a decay time constant of 500 μs, corresponding to a lower cutoff frequency of 300 Hz. The field waveforms are recorded with a sampling rate of 5 MS/s. More information on this electric field sensor can be found in [15].

### Meteorological data

Meteorological data in the Säntis region are available from ground-based and radar observations obtained by the Swiss Federal Office of Meteorology and Climatology (MeteoSwiss). A meteorological station owned by MeteoSwiss is set up at the bottom of the Tower. The measured data are collected by SwissMetNet, which is an automatic monitoring network of MeteoSwiss. The network currently comprises 160 measurement sites equipped with high-precision measurement instrumentation and state-of-the-art communication technology. In addition to the meteorological stations, MeteoSwiss also owns and operates a network of five C‑band Doppler polarimetric weather radars [16]. The network was recently renewed within the project Rad4Alp, which ended in 2016. The five systems have identical specifications and modes of operation. The scanning strategy consists of 20 horizontal scans with elevations ranging from −0.2 to 40° repeated every 5 min. Furthermore, vertical temperature profiles for the Säntis area are available by means of model-output soundings from MeteoSwiss. Using these profiles, one can extract the key environmental temperatures related to the convective microphysical and electrification processes.

### Temporary campaigns: Lightning Mapping Array and wideband interferometry

Other equipment that has been brought to the facility on a temporary basis include a lightning mapping array (LMA) belonging to the Lightning Research Group of the Polytechnic University of Catalonia (UPC), and a wideband interferometer, manufactured by New Mexico Tech.

A Lightning Mapping Array (LMA) is a discharge location system pioneered by D. E. Proctor [17–19] that can be used to produce 3D pictures of the lightning channel by locating radiation sources within and outside the cloud. The system operates by measuring the radiation from the discharges in the VHF band and calculating the location of the sources by way of the measured arrival times of the common signals at stations in different locations. Clustering algorithms [20–22] can be used to automatically identify lightning flashes from LMA data. In the Summer of 2017, the UPC’s Lightning Mapping Array [23, 24] was installed around the Säntis Tower. The installed LMA consisted of 6 stations. The LMA was operational during the months of July and August. A total of 20 lightning flashes were recorded and four were analyzed in detail [25].

**Fig. 13:**
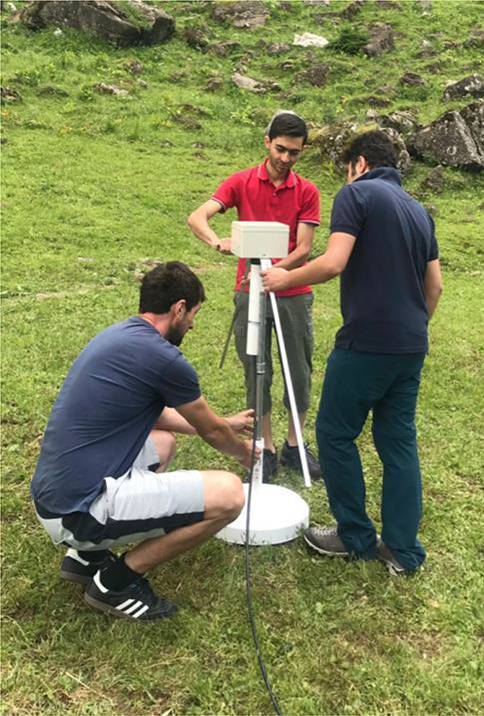
Picture of personnel from the HES-SO and the EPFL installing the antennas close to the Säntis Tower for use in the 2019 measurement campaign. From left to right: Antonio Sunjerga, Amirhossein Mostajabi, and Mohammad Azadifar

Interferometers use a larger bandwidth than LMAs (60 MHz for the interferometer and 6 MHz for the LMA). This makes the sensitivity of interferometric systems be higher than that of LMAs, although, as pointed out by Stock et al. [26], the increase in the sensitivity is not as high as one might expect since the antennas used in LMAs are more sensitive than those used in interferometers. Due to the better sensitivity of the interferometer, more breakdown sources are detected with those systems compared to LMAs. New Mexico Tech’s interferometer was installed near the Säntis in the Summer of 2019. Fig. 13 presents a picture of the installation of one of the VHF antennas of the interferometer. Interferometric data were obtained for 34 flashes to the tower, out of which 33 were upward negative flashes and 1 was an upward bipolar flash. The data are currently being analyzed.

## A selection of salient results

In the ten years since the current measurement instrumentation was first put in operation, the number of successfully recorded flashes has grown to nearly one thousand. Part of the measurement and analysis work that is currently being done at the Säntis is dedicated to the study of the conditions that would be conducive to the initiation of lightning in the presence of a Terawatt Laser beam. This work is being done in the context of the Laser Lightning Rod (LLR) H2020 European project [27]. The Terawatt Laser, which was installed next to the tower in 2021, was built for this specific application by one of the project partners, Trumpf AG, in southern Germany. The results obtained during the 2021 experimental campaign are currently under investigation.

A selection of the results obtained at the measurement facility is given in what follows.

### Negative flashes

The current data obtained since the put in operation of the facility constitute the largest dataset available to date for upward negative flashes. The analysis has contributed to the characterization of the different types of currents and electric field pulses that are associated with this type of flash. This has led to new models for the physical processes involved in their generation and it has corroborated the statistical similarities between pulses in upward and downward lightning (e.g., [28, 29]).

### Characterization of positive flashes

Although positive lightning flashes are considerably less frequent than negative flashes, they are of great practical importance since they are associated with especially high currents and charge transfer values and therefore represent a higher risk regarding lightning-originated fires and the protection of power and electronic equipment.

Measurements made at the Säntis tower resulted in the characterization of two distinct types of upward positive flashes based on the current measured at the base of the channel [30]. These two types are similar to those observed by Berger [31]. Type‑I upward lighting flashes are characterized by the presence of a large unipolar current pulse, typically preceded by bursts of fast pulses superimposed on a continuous current. Type-II flashes, on the other hand, differ from Type‑I flashes in the absence of the large unipolar pulse but they exhibit longer durations and higher amounts of charge transfer.

### Propagation over irregular terrain

**Fig. 14:**
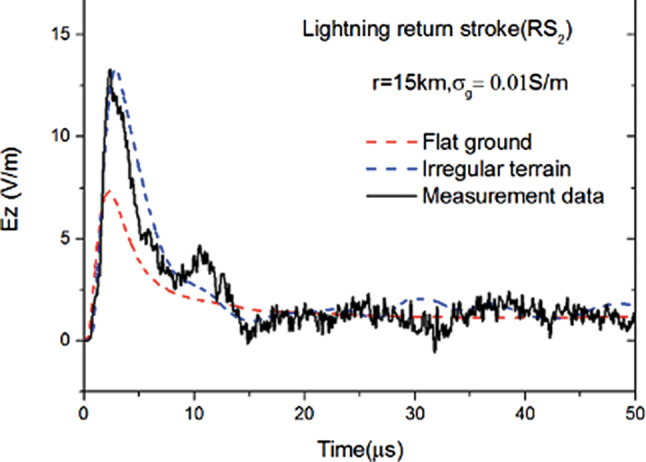
Vertical electric field at 15 km associated with a return stroke in a flash to the Säntis. Solid line: measured waveform. Red dashed line: simulated waveform assuming a flat ground. Blue dashed line: simulated waveform taking into account the terrain profile. Ground parameters: σ_g_ = 0.01 S/m and ε_rg_ = 10. (Adapted from [32])

Simultaneous field and current measurements together with FDTD simulations have allowed the study of propagation of the radiation from lightning over mountainous terrain taking advantage of the Alpine region where the Säntis tower is located. The effect of the rough terrain on the fields can be observed in Fig. 14, from which it can be concluded that the assumption of a flat terrain can lead to errors in the peak values of the fields of the order of 40% and that it is possible to obtain excellent agreement between measured and simulated fields by using a 2D-FDTD approach [32]. This result has important implications for the remote estimation of the lightning currents in lightning location systems.

### Performance of the European Lightning Detection Network for Upward Flashes

**Fig. 15:**
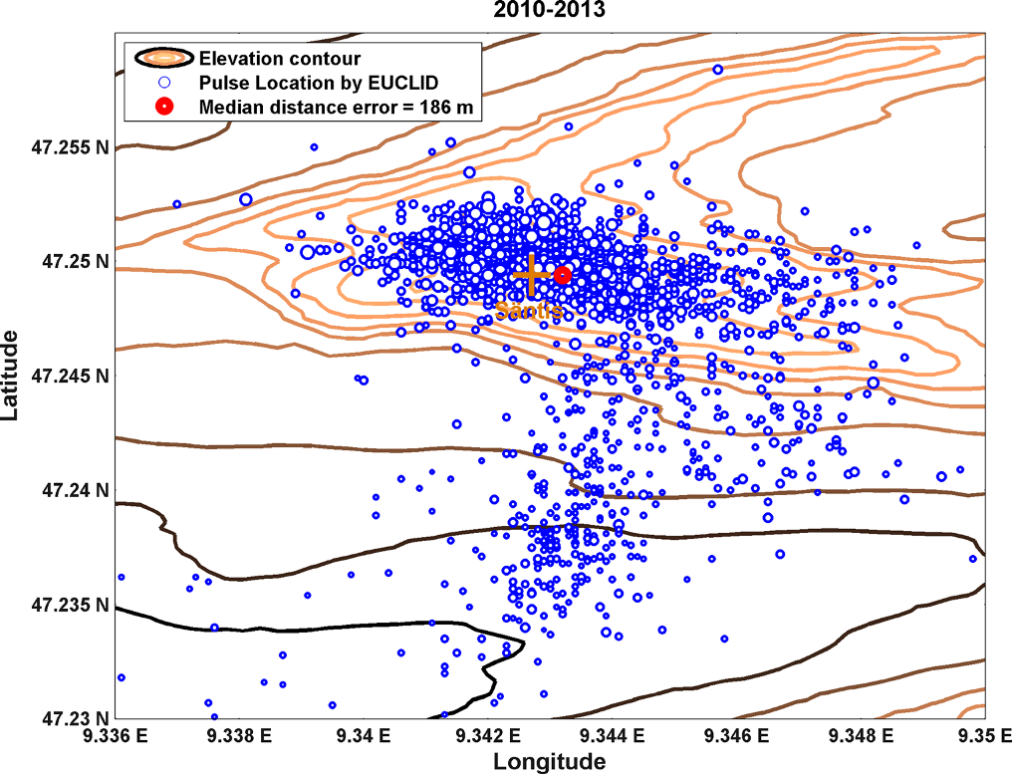
Plot of EUCLID pulse locations for upward negative flashes in the region of the Säntis tower from 2010 to 2013. The size of the circles is proportional to the current peak measured at the Säntis. The length and width of the shown area are respectively 3.34 and 1.06 km. (Adapted from [33])

The availability of the channel-base current and the exact lightning strike location make instrumented towers an excellent ground-truth for the evaluation of the performance of lightning location systems. A performance analysis of the European lightning detection network (EUCLID) was performed using data obtained on lightning currents measured at the Säntis Tower in 2016. The performance of the EUCLID lightning detection network was evaluated in terms of detection efficiency, location accuracy and peak current estimates for upward flashes [33]. The median location error was found to be 186 m. The detection efficiency for upward flashes with pulses larger than 2 kA was estimated to be 97%. The detection network overestimated the currents by a factor of 1.8 because of the field enhancement due to the presence of the mountain, as demonstrated by full wave simulations of the region [32]. Fig. 15 shows a map of the locations of pulses detected by the EUCLID system corresponding to strikes to the Säntis tower. The dots in the figure represent the locations given by the EUCLID system. The cluster of locations with larger errors to the south of the tower were shown to be associated with pulses of lower amplitude, indication that the location accuracy is dependent on the amplitude of the current peaks.

### Lightning-ionosphere interactions

**Fig. 16:**
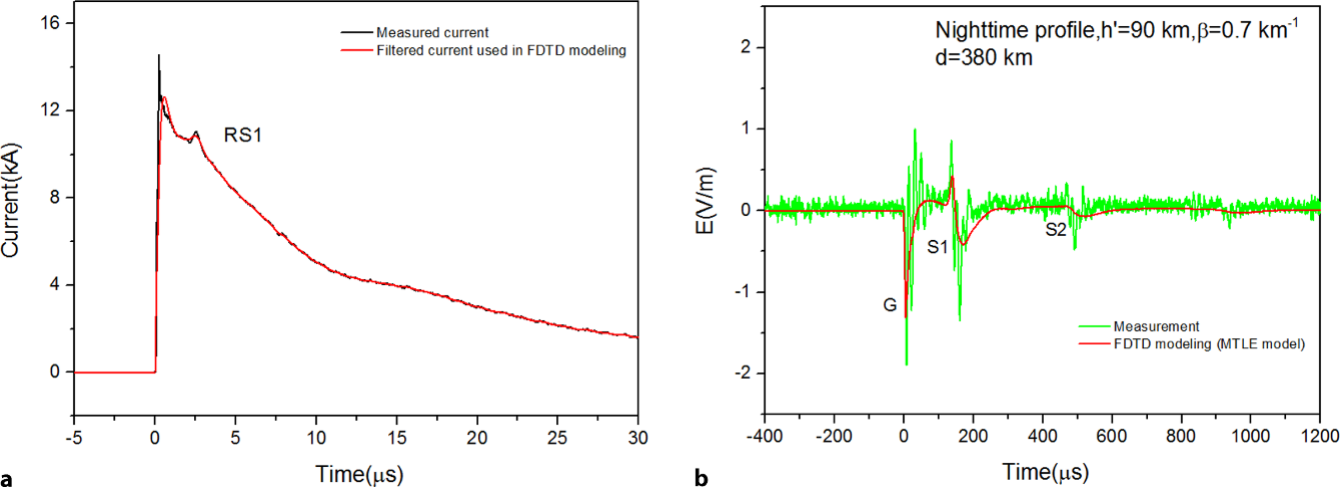
Current and electric field waveforms produced by the first return stroke of a nighttime upward flash that occurred on 21 October 2014 at 20:23:22. **a** Measured current (*black*) and 2‑MHz low-pass filtered (*red*) current used in FDTD simulations. **b** Measured (*green*) and simulated (*red*) E‑field waveforms at 380 km. Adapted from [34]

The electromagnetic fields from distant lightning flashes constitute a valuable source of information in the study of the properties of the ionosphere. Simultaneous measurements of currents from upward lightning strikes to the Säntis tower and of wideband electric field waveforms at 380 km constitute the first ever measurements of that type that feature ionospheric reflections for natural upward flashes in the field waveforms. The 380 km field measurements represent, in addition, the longest distance at which natural upward lightning fields have been measured simultaneously with their causative currents [34]. Intervals between the ground wave field signatures and those of the skywaves were used to evaluate ionospheric reflection characteristics during daytime and nighttime based on the so-called zero-to-zero and peak-to-peak methods. Fig. 16 shows a plot of the current and electric field waveforms produced by the first return stroke of a nighttime upward flash that occurred on 21 October 2014. Fig. 16b shows the comparison between measured and simulated fields at 380 km.

Li et al. [35] investigated using a full-wave Finite-Difference-Time-Domain model the effect of the Earth-ionosphere waveguide structure and medium parameters, including the effect of the ionospheric cold plasma characteristics, the effect of the Earth curvature, and the propagation effects over mountainous terrain. The obtained results were validated against simultaneous experimental data of lightning currents measured at the Säntis Tower and electric fields measured in Neudorf, Austria. It was shown that both the time delays and amplitudes of the lightning electromagnetic fields at 380-km distance can be strongly affected by the ionospheric electron density profile, the mountainous terrain, and the Earth curvature.

Mostajabi et al. [36] presented and discussed simultaneous records of current and electric fields 380 km from the strike point associated with an upward bipolar flash initiated from the Säntis Tower. The flash contained 23 negative strokes and one positive stroke. The intervals between the groundwave and skywave arrival times were used to estimate ionospheric reflection heights for the negative return strokes using the so-called zero-to-zero and peak-to-peak methods. It was found that the ratio of the peak field to the current peak is about two times smaller for the positive pulse compared to negative pulses. This difference in the amplitudes can be attributed to a lower return stroke speed for the positive stroke compared to that for negative strokes, and also to the fact that the enhancement of the electric field due to the presence of the tower and the mountain might be more significant for negative pulses, which are characterized by faster risetimes compared to the positive one.

### Validation of single-sensor lightning location method

**Fig. 17:**
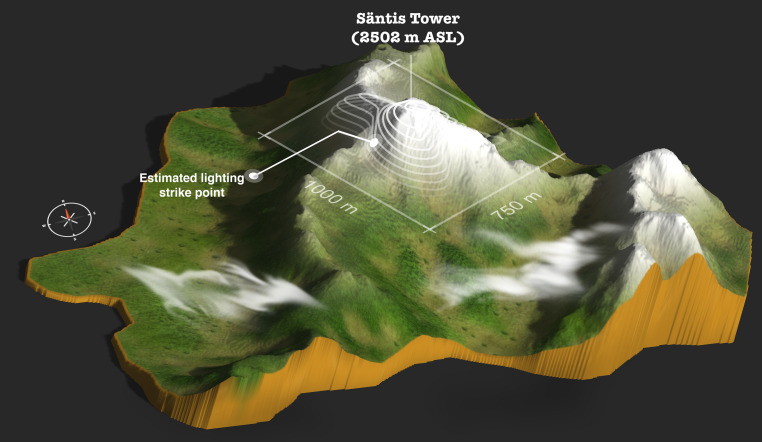
Experimental validation result for the EMTR/ML single-sensor location method. (Adapted from [37])

A combination of electromagnetic time reversal (EMTR) and Machine Learning was proposed for the first time by Mostajabi et al. [37] to localize electromagnetic sources using a single electric field sensor that relies only on the presence of scatterers such as mountains. Mostajabi et al. trained and tuned their Machine Learning model using simulation results and tested it using the data from the European Cooperation for Lightning Detection (EUCLID) for lightning in the region of the Säntis. The location error was about 300 m, comparable to current commercial lightning location systems that use multiple sensors. Mostajabi et al. tested the location technique experimentally using an upward lightning flash that occurred at the Säntis Tower and, as the single-site recording, the associated electric field 14.7 km away at Herisau. The tower and the estimated lightning strike point are shown in Fig. 17. The calculated strike is 148 m away from the Säntis Tower.

### New type of bipolar flash

Bipolar flashes transfer charge of both polarities to ground. Rakov [38] classified upward bipolar flashes into three categories: Category I flashes, which present a polarity reversal during the initial continuous current with a possible no-current interval between the two polarities; Category II flashes, characterized by an initial-stage current and the following return stroke or strokes of different polarity; and Category III flashes, which contain return strokes of opposite polarities.

**Fig. 18:**
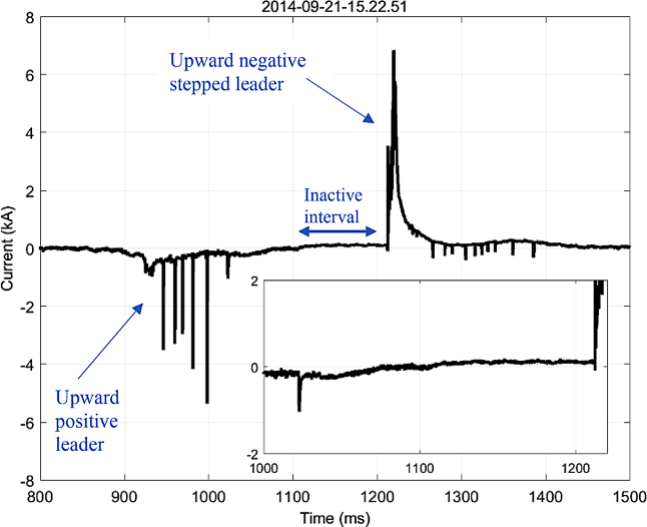
Bipolar flash that occurred on 21 September 2014. An expanded view of the inactive steady current is shown in the inset. (Adapted from [39])

A study of current waveforms associated with 13 bipolar lightning flashes recorded at the Säntis Tower over a four and half year period was presented by Azadifar et al. [39]. They observed that two of ten Category‑I flashes with no return strokes consisted of a sequence of two upward discharges of different polarity initiated from the tower within tens of milliseconds of each other, which they interpreted as a succession of two opposite-polarity flashes with a very short inter-flash interval. As this type of Category‑I flash had not been observed before, and given the potential implications of such type of flash for lightning protection, Azadifar et al. argued for the expansion of the traditional classification by splitting Category I to include two subcategories, one exhibiting a polarity change in the initial continuous current with no evidence of two separate channels, and another in which separate, consecutive flashes less than 100 ms apart compose the overall flash. An example of the new type of bipolar flash observed at the Säntis tower is shown in Fig. 18.

### Modeling of lightning processes

Data from the Säntis tower have been used to further our understanding of processes in upward lightning and to model those processes.

An important example with implications for lightning protection is the study of current pulses in upward lightning currents. Upward negative lightning exhibits pulses associated with three charge transfer modes: The return stroke mode of charge transfer, the M component mode of charge transfer, and the mixed mode of charge transfer. He et al. [40, 41] used channel-base lightning currents and simultaneously measured electric fields 15 km from the tower to show that both, mixed mode (MM) pulses during the Initial Continuous Current (ICC) and return strokes (RSs) that occur after the cessation of the ICC can be simulated using the Modified Transmission Line Model with Exponential Decay (MTLE), in agreement with the assumption that the mode of charge transfer to the ground of MM pulses is similar to that of RSs. He at al. also showed that M component pulses that happen during the continuing current (MCs) of some return strokes and M‑ICC pulses superimposed on the ICC can be simulated using the guided wave model of Rakov et al. [42], lending further support to the similarity between the physical processes giving rise to M‑ICC pulses and classical MCs.

Other work based on measurements made at the Säntis tower has helped advance our understanding of the origin of fast pulses observed in the radiated electric field from M components, whose currents are orders of magnitude slower [43].

### Observations of upward flashes using Lightning Mapping Arrays

Using simultaneous measurements of the electric field, channel base currents at the Säntis tower and data from a Lightning Mapping Array (LMA) belonging to the Polytechnical University of Catalonia that was installed in the Säntis tower area in 2017, Sunjerga et al. [25] analyzed 20 upward flashes that occurred at the tower and determined that four of them were of the other-triggered (OT) type. OT flashes are characterized by the presence of (and are thought to be triggered by) preceding cloud or downward cloud-to-ground lightning activity in the area of the upward flash. Sunjerga et al. observed that only one of the four flashes would have been classified as an OT flash if only data from the European Lightning Detection Network (EUCLID) had been used. Indeed, based on the analysis of the LMA data, the other 3 flashes were observed to be preceded by nearby activity that overlapped with the upward flash in time or that preceded it by at most 300 ms and that should therefore be classified as OT flashes.

**Fig. 19:**
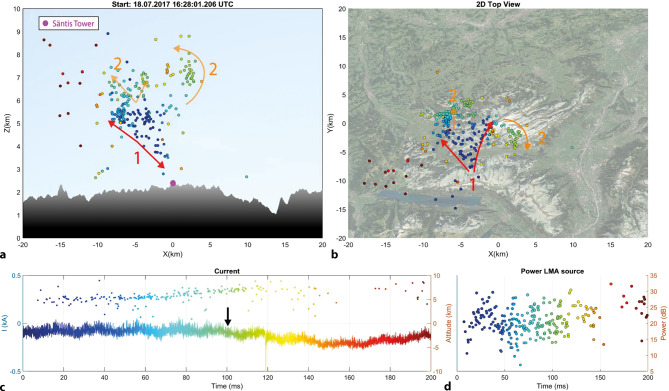
Initial stage of an upward negative flash initiated from the Säntis tower recorded on 18.07.2017 at 16:28:01 UTC. In the upper left panel , the location of the tower is shown with a purple marker and the LMA VHF sources are shown with time-color-coded circle markers. **a** 2D view of Z vs. X, **b** 2D view of X vs. Y, **c** current with VHF sources superimposed (1 kHz low-pass filter applied), **d** power vs. time for the VHF sources. Note that the colors of the arrows in (**a**) and (**b**) were selected for better contrast and do not bear a relation to the color-code used for timing. The start of the time axis corresponds to the time given in the title of subplot (**a**). The colored arrows show the development of in-cloud leaders. Adapted from [25]

An example of the initial stage of an upward flash mapped with the LMA in the 2017 campaign, including a plot of the direct current measured at the Säntis tower is shown in Fig. 19.

### Meteorology

Figueras et al. [16] applied the MeteoSwiss Thunderstorms Radar Tracking (TRT) algorithm in the area surrounding the Säntis mountain during the 2017 LMA lightning measurement campaign at the Säntis to analyze the lightning production of convective cells. They investigated the relationship between the intra-cloud (IC) and cloud-to-ground (CG) activity and the cell severity and they proposed a new metric to quantify lightning intensity: the rimed-particle column (RPC) height and base altitude. Figueras et al. showed that the RPC metric is a promising predictor of lightning activity, particularly for IC flashes. In [44], Figueras et al. related LMA sources data with collocated radar data in order to characterize the main features of both the flash origin and its propagation path. They showed that polarimetric weather radar data can be helpful in determining regions where lightning is more likely to occur but that lightning climatology and/or knowledge of the orography and man-made structures is also relevant.

### Potential impact on lightning standards

Upward lightning data obtained at the Säntis tower support the need to revisit current waveforms used in standardized aircraft and wind turbine blade testing. It has been argued [45] that waveforms based on upward flashes may be better suited as a basis for testing than the currently used tests, which are based on downward lightning and on airborne measured current bursts.

## Conclusions and future perspectives

We presented the Säntis Tower Lightning Research Facility that includes the Säntis Tower, which has been hit by lightning consistently 100 or more times a year for the past decade. This one-of-a-kind, life-size laboratory for experimental lightning research is currently being exploited by the EMC laboratory of the Swiss Federal Institute of Technology, Lausanne and the Advanced Communication Systems Group of the University of Applied Sciences Western Switzerland, Yverdon-les-Bains.

We described the instrumentation at the tower for direct current and current derivative measurements, fast and slow electromagnetic fields 20 m from the tower, standard and high-speed video in the tower vicinity, and vertical electric field measurements at 380 km. We also described the lightning mapping and high-energetic radiation instruments that have been or are being used on a temporary basis at the facility. We presented salient results obtained from the analysis of the data recorded during the first decade of operation.

The aim of the Säntis experimental facility is to improve our understanding of lightning discharges, particularly their initiation mechanisms and their development. To achieve this task, we will enhance the instrumentation of the Säntis tower and its surroundings. We will develop a multi-band spectrum lightning measurement station from VLF to LF, VHF, microwaves, light, and X‑rays. Sources involved in the initiation of the discharge will be identified and located using interferometric and ToA Lightning Mapping Array techniques. The new measuring equipment will be synchronized with the existing sensors and it will be integrated into the remote monitoring, data transfer and storage system deployed at the Säntis tower. The foreseen experimental data and theoretical investigations will allow a better understanding of the characteristics of upward flashes from tall structures and the mechanism of their initiation, which is essential for the design of lightning protection systems for tall structures including wind turbines.

## References

[1] Uman MA (1987). The lightning discharge.

[2] Early Metrics Key trends and challenges for the smart grid market. https://earlymetrics.com/key-trends-and-challenges-for-the-smart-grid-market/ (Created 16 Mar 2020). Accessed 17 Dec 2020

[3] Price C (2009). Will a drier climate result in more lightning?. Atmos Res.

[4] Asfur M, Silverman J, Price C (2020). Ocean acidification may be increasing the intensity of lightning over the oceans. Sci Rep.

[5] Rakov VA (2013). Lightning parameters for engineering applications.

[6] Berger K, Anderson RB, Kroninger H (1975). Parameters of lightning flashes. Electra.

[7] McCann T (2008). The geology of central Europe: mesozioc and cenozoic.

[8] Romero C, Rachidi F, Paolone M, Rubinstein M “Statistical distributions of lightning currents associated with upward negative flashes based on the data collected at the Säntis (EMC) tower in 2010 and 2011,” IEEE Trans. Power Deliv., vol. 28, no. 3, pp. 1804–1812, 2013. http://ieeexplore.ieee.org/abstract/document/6532314/. Accessed 29 Mar 2017

[9] Baum CE (1964). Maximizing frequency response of a b-dot loop. Sens Simul Notes.

[10] Baum C, Breen E, Giles J, O’neill J, Sower G (1978). Sensors for electromagnetic pulse measurements both inside and away from nuclear source regions. IEEE Trans Antennas Propag.

[11] Romero C (2010). Measurement of lightning currents using a combination of Rogowski coils and B-Dot sensors.

[12] Romero C (2012). A system for the measurements of lightning currents at the Säntis tower. Electr Power Syst Res J.

[13] Azadifar M, Paolone M, Pavanello D, Romero C, Rachidi F, Rubinstein (2014). An update on the instrumentation of the Säntis Tower in Switzerland for lightning current measurements and obtained results.

[14] Azadifar M (2014). An update on the charaterictics of positive flashes recorded on the Säntis Tower. Lightning Protection (ICLP).

[15] Pichler H, Diendorfer G, Mair M (2010). Some parameters of correlated current and radiated field pulses from 339 lightning to the Gaisberg tower. IEEJ Trans Electr Electron Eng.

[16] Figueras i Ventura J (2019). Analysis of the lightning production of convective cells. Atmos Meas Tech.

[17] Proctor DE (1971). Hyperbolic system for obtaining VHF radio pictures of lightning. J Geophys Res.

[18] Proctor DE, Uytenbogaardt R, Meredith BM (1988). VHF radio pictures of lightning flashes to ground. J Geophys Res Atmos.

[19] Proctor DE (1981). VHF radio pictures of cloud flashes. J Geophys Res Oceans.

[20] Fuchs BR (2015). Environmental controls on storm intensity and charge structure in multiple regions of the continental United States. J Geophys Res Atmos.

[21] McCaul EW, Goodman SJ, LaCasse KM, Cecil DJ (2009). Forecasting lightning threat using cloud-resolving model simulations. Weather Forecast.

[22] MacGorman DR (2008). TELEX the thunderstorm electrification and lightning experiment. Bull Am Meteorol Soc.

[23] Thomas RJ (2004). Accuracy of the lightning mapping array. J Geophys Res.

[24] Rison W, Thomas RJ, Krehbiel PR, Hamlin T, Harlin J (1999). A GPS-based three-dimensional lightning mapping system: Initial observations in central New Mexico. Geophys Res Lett.

[25] Sunjerga A (2019). LMA observations of upward lightning flashes at the Säntis Tower initiated by nearby lightning activity. Electr Power Syst Res.

[26] Stock MG (2014). Continuous broadband digital interferometry of lightning using a generalized cross-correlation algorithm. J Geophys Res Atmos.

[27] Produit T (2020). The laser lightning Rod project. Eur Phys J Appl Phys.

[28] Azadifar M (2016). Fast initial continuous current pulses versus return stroke pulses in tower-initiated lightning. J Geophys Res Atmos.

[29] He L (2018). An analysis of current and electric field pulses associated with upward negative lightning flashes initiated from the Säntis tower. J Geophys Res Atmos.

[30] Romero C, Rachidi F, Rubinstein M, Paolone M, Rakov VA, Pavanello D (2013). Positive lightning flashes recorded on the Säntis tower from May 2010 to January 2012. J Geophys Res Atmos.

[31] Berger K (1978). Blitzstorm-Parameter von Aufwartsblitzen. Bull Schweiz Electrotech.

[32] Li D (2016). On lightning electromagnetic field propagation along an irregular terrain. IEEE Trans Electromagn Compat.

[33] Azadifar M (2016). Evaluation of the performance characteristics of the European Lightning Detection Network EUCLID in the Alps region for upward negative flashes using direct measurements at the instrumented Säntis Tower. J Geophys Res Atmos.

[34] Azadifar M (2017). Analysis of lightning-ionosphere interaction using simultaneous records of source current and 380 km distant electric field. J Atmos Sol Terr Phys.

[35] Li D (2019). The propagation effects of lightning electromagnetic fields over mountainous terrain in the earth-ionosphere waveguide. J Geophys Res Atmos.

[36] Mostajabi A (2019). Analysis of a bipolar upward lightning flash based on simultaneous records of currents and 380-km distant electric fields. Electr Power Syst Res.

[37] Mostajabi A, Karami H, Azadifar M, Ghasemi A, Rubinstein M, Rachidi F (2019). Single-sensor source localization using electromagnetic time reversal and deep transfer learning: application to lightning. Sci Rep.

[38] Rakov VA (2003). A review of positive and bipolar lightning discharge. Bull Am Meteor Soc.

[39] Azadifar M, Rachidi F, Rubinstein M, Rakov VA, Paolone M, Pavanello D (2016). Bipolar lightning flashes observed at the Säntis Tower: Do we need to modify the traditional classification?. J Geophys Res Atmos.

[40] He L (2019). Electromagnetic fields associated with the M-component mode of charge transfer. J Geophys Res Atmos.

[41] He L (2019). Characteristics of different charge transfer modes in upward flashes inferred from simultaneously measured currents and fields. High Volt.

[42] Rakov VA, Thottappillil R, Uman MA, Barker PP (1995). Mechanism of the lightning M component. J Geophys Res.

[43] Azadifar M, Rubinstein M, Li Q, Rachidi F, Rakov V (2019). A new engineering model of lightning M component that reproduces its electric field waveforms at both close and far distances. J Geophys Res Atmos.

[44] Figueras i Ventura J (2019). Polarimetric radar characteristics of lightning initiation and propagating channels. Atmos Meas Tech.

[45] Smorgonskiy A, Rachidi F, Rubinstein M, Korovkin NV, Vassilopoulos AP (2017). Are standardized lightning current waveforms suitable for aircraft and wind turbine blades made of composite materials?. IEEE Trans Electromagn Compat.

